# Extending Transition-Metal Carbo-Chalcogenides to Tellurium with Nb_2_CTe_2_

**DOI:** 10.1021/acsomega.6c02686

**Published:** 2026-07-29

**Authors:** Yiftach Kushnir, Mark Baranov, Bar Favelukis, Asaf Nitsan, Or Messer, Alexander Upcher, Vladimir Ezersky, Nitzan Maman, Barak Ratzker, Maxim Sokol

**Affiliations:** † Department of Materials Science and Engineering, 26745 Tel Aviv University, P.O.B 39040, Ramat Aviv, Tel Aviv-Yafo 6997801, Israel; ‡ Ilse Katz Institute for Nanoscale Science and Technology, 26732 Ben-Gurion University of the Negev, P.O.B 653, Beer-Sheva 8410501, Israel; § Max Planck Institute for Sustainable Materials, Max-Planck-Str. 1, Düsseldorf 40237, Germany

## Abstract

Transition-metal carbo-chalcogenides (TMCCs) are expanding the structural and chemical diversity of layered van der Waals (vdW) materials by bridging transition-metal dichalcogenides (TMDs) and transition-metal carbides (MXenes). This study reports the experimental realization of Nb_2_CTe_2_, the first tellurium-based TMCC phase. Using a sealed-ampule reactive synthesis strategy, Nb_2_CTe_2_ was obtained as the predominant phase (78.0 wt %). X-ray diffraction, scanning electron microscopy, together with atomic-resolution scanning transmission microscopy structural and chemical analysis, confirm a layered trigonal structure (*P*3*®m*1) and lamellar flake-like morphology composed of Nb–C slabs capped by Te layers. High-angle annular dark-field scanning transmission electron microscopy-derived interlayer separation, together with the previously predicted low delamination energy, supports weak out-of-plane vdW coupling. Long-term ambient exposure revealed limited chemical stability in air, with near-complete loss of the Nb_2_CTe_2_ phase, increased diffuse scattering, and oxidative degradation over time. This work expands the experimentally accessible TMCC family to Te-containing compositions and provides a foundation for future studies of heavy-chalcogen TMCCs and their two-dimensional derivatives.

## Graphical abstract

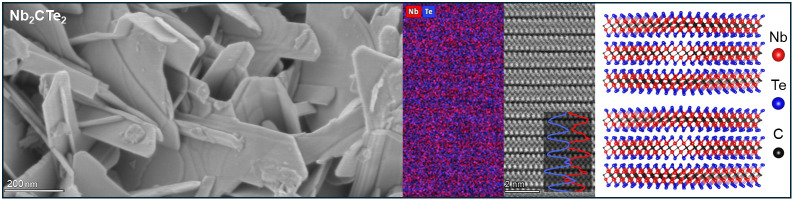

## Introduction

Transition-metal carbo-chalcogenides (TMCCs) have recently emerged as a distinct class of layered materials that structurally bridge two well-established families of low-dimensional compounds, namely transition metal dichalcogenides (TMDs) and transition metal carbides and/or nitrides (MXenes).^1,2^ TMDs consist of a transition metal sandwiched between two chalcogen layers, forming layered structures held together by van der Waals (vdW) interactions.^3^ MXenes comprise transition-metal carbide or nitride layers characterized by high conductivity and tunable surface chemistry.^4^ TMCCs are described by the formula M_2_CCh_2_, in which carbide-based layers are capped by chalcogen layers. In this architecture, the central metal layer of a TMD is effectively replaced by a MXene-like carbide slab.^5^

The first experimentally realized TMCC phases were reported in the 1970s with the synthesis of Ta_2_CS_2_,^6^ followed by the subsequent realization of Nb_2_CS_2_ in the early 1990s,^7^ and more recently by additional selenium-based TMCCs, including Ta_2_CSe_2_ ^8^ and Nb_2_CSe_2_.^9^ Both sulfur- and selenium-based TMCCs have been reported to exhibit metallic electronic behavior,^10^ high electrical conductivity,^11^ and promising electrochemical and catalytic performance.^8,12–14^ In addition to their reported properties, the intrinsic structure of TMCCs is of great interest. Consistent with the nature of the TMDs, first-principles calculations have predicted TMCCs to be intrinsically vdW-bonded layered solids,^15^ with delamination energies comparable to those of other exfoliable vdW solids (∼47–56 meV/atom).^16^ Consequently, experimental studies have validated these predictions, with Majed et al.^1^ first demonstrating the delamination of TMCCs (Nb_2_CS_2_ and Ta_2_CS_2_) into single- or few-layer 2D sheets, and more recently Loni et al.^8^ extending this approach to Ta_2_CSe_2_, where the delaminated material exhibited enhanced hydrogen evolution reaction activity compared to its bulk counterpart. Importantly, this delamination represents a direct route to MXene-like two-dimensional materials without the need to first synthesize and chemically etch a parent three-dimensional MAX phase, distinguishing TMCCs from conventional MXene systems.^17^

Despite growing interest in TMCCs, only a limited number of phases have been synthesized, with Te-based compounds remaining unrealized to date.^18^ Notably, Te-based compounds continue to attract interest due to the diverse electronic characteristics reported among heavier chalcogen systems.^19–21^ Layered or van der Waals tellurides are additionally of interest because weak interlayer bonding can confine charge carriers within atomic planes, thereby enabling effective tuning of the electronic structure in reduced dimensions.^22,23^ In this context, Nb_2_CTe_2_ expands the TMCC family into Te-containing compositions and offers a new layered framework for future structural and property studies.

A recent density functional theory (DFT)-based computational screening study by Helmer et al.^15^ systematically evaluated chalcogen-terminated TMCCs and identified several compositions with negative formation enthalpy. All thermodynamically stable TMCC compositions identified in that study contained Group V transition metals (V, Nb, or Ta), identifying Group V chemistry as a common feature of all predicted stable TMCC compositions. Notably, this study predicted the thermodynamic stability of Ta_2_CSe_2_, which was subsequently synthesized in an ensuing study.^8^ Within the same DFT study, Nb_2_CTe_2_ was also predicted to have the most negative formation enthalpy among the screened Te-based TMCC compositions indicating higher thermodynamic stability relative to competing phases in the ternary M–C–Te systems (M = transition metal). Guided by this theoretical prediction, this work presents the experimental realization of Nb_2_CTe_2_ through a reactive synthesis strategy, establishing it as the first Te-based TMCC phase.

## Methods

### Synthesis

Elemental powders of Nb (Sigma-Aldrich, 325 mesh, 99.8%), C (Thermo Scientific, 325 mesh, 99%), and Te (Thermo Scientific, 200 mesh, 99.8%) were used as starting materials for the synthesis of Nb_2_CTe_2_. We note in passing that previous TMCC syntheses have employed more indirect precursor routes, including Fe- and Cu-containing compounds used to stabilize intermediate intercalated phases, or reagents such as C_6_Cl_6_ introduced to facilitate phase formation.^11^ In the present case, owing to the low melting point and high volatility of Te, which restrict direct high-temperature synthesis in open systems, the reaction was carried out in a sealed quartz ampule. Stoichiometric amounts of Nb, C, and Te (Nb:C:Te = 2:1:2) were homogenized using an agate mortar and pestle, followed by additional mixing in a rolling tumbler at 180 rpm for 12 h. The powder mixture was compacted into 10 mm diameter pellets under a uniaxial pressure of 200 MPa. The pellets were sealed in a quartz ampule under a vacuum of ∼680 Torr after Ar purging and subsequently heat-treated at 1200 °C for 2 h in a muffle furnace using heating and cooling rates of 10 °C/min. The selected temperature is consistent with that used for previously reported experimentally realized TMCC phases.^6–9^ After cooling to room temperature, the ampule was opened and the reacted pellets were readily pulverized for further characterization. The reported synthesis conditions were selected after evaluating varied precursor atomic ratios, temperatures, and dwell times.

### Characterization

X-ray diffraction (XRD) was used for phase identification and structural characterization using a PANalytical AERIS diffractometer equipped with a Cu Kα X-ray source (λ = 1.5406 Å). Diffraction patterns were collected over a 2θ range of 5°–85°, with a step size of 0.01086° and a scanning rate of 0.0557 °/s. Lattice parameters, symmetry determination, and structural refinement were carried out using the GSAS-II software package (version 55818, 2025 release).^24^ Background intensities were modeled using Chebyshev polynomial functions, and Rietveld refinements were performed using a full-matrix least-squares approach with pseudo-Voigt peak profile functions. Final refinements accounted for instrumental broadening, sample-related effects, and atomic positional parameters. The relative crystalline and amorphous content within the sample was estimated using the AMORPH program by utilizing a Bayesian statistical approach to fit both the narrow crystalline components and the broad amorphous component.^25^ The range for amorphous content characterization was set between 2θ = 20°–50° as it is deemed ideal for inclusion of both types of components. The narrow peaks were fitted utilizing a Gaussian distribution, while the amorphous peak was fitted utilizing Pseudo-Voigt function. Graphic illustrations of the Nb_2_CTe_2_ TMCC phase were created with the VESTA software package (2025, ver. 3.90.5a).^26^ The final refinement cycle included the atomic, instrumental, and sample parameters.

Microstructural characterization was performed by high-resolution scanning electron microscopy (SEM) using ZEISS Gemini 300 and FEI Helios G4 UC instruments operated at an accelerating voltage of 5 kV. Given that the synthesized material contained secondary phases, regions for FIB lamella preparation were first screened based on morphology and local EDS analysis. Transmission electron microscopy (TEM) lamellae were prepared by focused ion beam (FIB) milling using the FEI Helios G4 UC system. Prior to milling, a protective carbon coating was deposited over the region of interest to minimize ion-beam damage during lamella preparation. TEM analysis was conducted in scanning transmission electron microscopy (STEM) mode using a Cs-corrected Thermo Fisher Scientific Spectra 200 microscope operated at 200 kV. A high-angle annular dark-field (HAADF) detector was used for imaging and energy-dispersive X-ray spectroscopy (EDS) spectra were acquired using a Super-X silicon drift detector (SDD), with data processing carried out using Velox software (version 3.12). TEM images were processed using high-pass and radial Wiener filtering.

## Results and Discussion

The synthesized powder product was examined to confirm the TMCC phase structure and chemistry. Figure 1 presents the XRD pattern of the synthesized material together with the corresponding Rietveld refinement. The diffraction pattern displays dominant low-angle reflections at 2θ = 8.97° and 17.95°, which are indexed to the (001) and (002) basal planes, respectively, indicating a layered crystal structure with pronounced periodicity along the stacking direction. Consistent with other reported TMCC phases,^6–9^ Nb_2_CTe_2_ adopts a trigonal structure with *P*3̅*m*1 symmetry, characterized by refined lattice parameters *a* = 3.4831 Å and *c* = 9.8844 Å. Relative to previously reported S- and Se-based TMCC phases, which exhibit *c* lattice parameters of ∼8.5–9.0 Å,^6–9^ Nb_2_CTe_2_ shows expanded lattice parameters, particularly along the stacking direction, consistent with incorporation of the larger Te atom within the layered structure. This behavior follows the predicted trend of increasing lattice dimensions with increasing chalcogen size across the TMCC family,^15^ while preserving the characteristic trigonal TMCC framework. An illustration of the crystal structure is shown alongside the XRD pattern in Figure 1, highlighting the layered nature of Nb_2_CTe_2_ and its similarity to vdW multilayered MXene-like structures. Structural refinement using the Rietveld method resulted in a refinement indicator of *R*_wp_ = 7.63%, indicating good agreement between the experimental data and the proposed structural model. Additional refinement details, including refined crystallographic parameters and Wyckoff positions, are provided in Tables 1 and 2, respectively.

**Figure 1:**
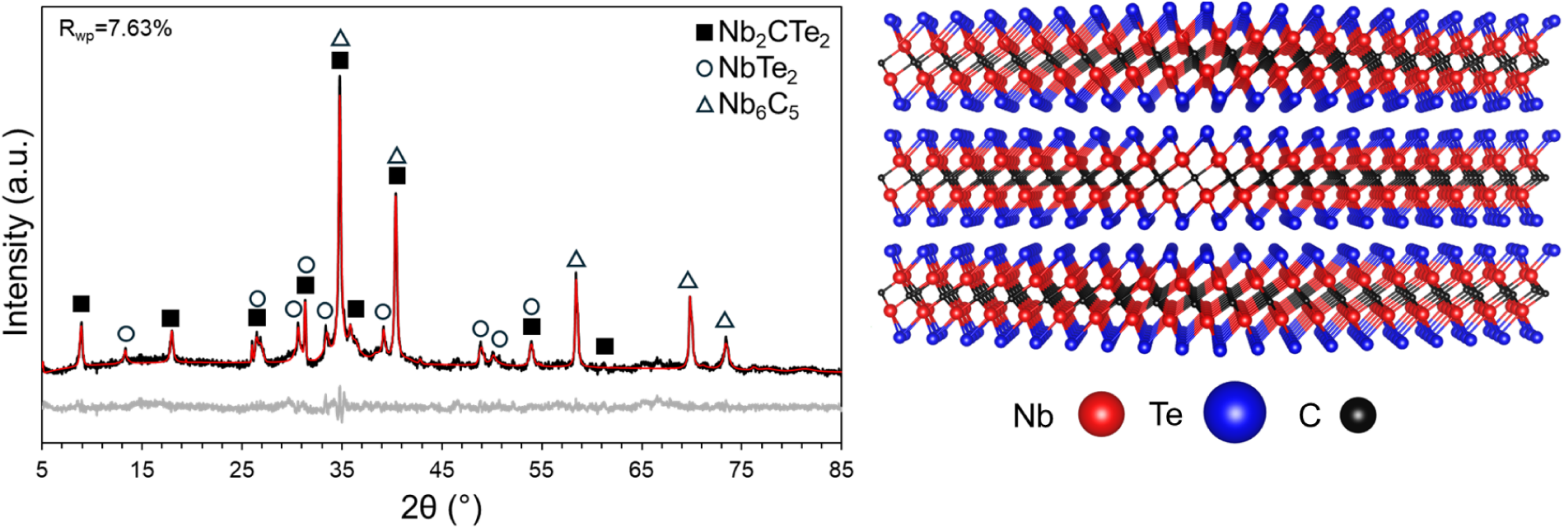
XRD pattern of the synthesized Nb–C–Te system with corresponding Rietveld refinement analysis. The experimental data, calculated pattern, and difference curve are shown as black, red, and gray lines, respectively. Refinement indicator (*R*_wp_ = 7.63%) confirms that the Nb_2_CTe_2_ TMCC phase is the predominant synthesis product (78.0 wt %), along with Nb_6_C_5_ (15.5 wt %) and NbTe_2_ (6.5 wt %). A schematic illustration of the Nb_2_CTe_2_ crystal structure viewed along [100] is shown on the right.

**Table 1:** Crystal Data and Structure Refinement Details of the Nb_2_CTe_2_ TMCC Phase

Chemical Formula	Nb_2_CTe_2_
Molar mass (g/mol)	453.02
Crystal system, space group	Trigonal, *P*3*®m*1 (164)
a, c lattice parameters (Å)	3.4831, 9.8844
Unit cell volume (Å^3^)	101.185
Formula units-Z	1
Density (g/cm^3^)	7.435
Refinement indicator *R*_wp_ (including all data)	7.63%
Goodness of fit	2.09
CCDC #	2521070

**Table 2:** Wyckoff Positions and Occupancies within the Unit Cell of Nb_2_CTe_2_ TMCC Phase

Atom designation	Wyckoff position (coordinates)	Occupancy
Nb1 (Nb)	2d (2/3,1/3, 0.85197)	1
C1 (C)	1a (0, 0, 0)	1
Te1 (Te)	2d (1/3, 2/3, 0.63312)	1

The reactive synthesis approach, involving the reaction of elemental precursors in a sealed quartz ampule, yielded Nb_2_CTe_2_ as the predominant phase. Quantitative phase analysis based on Rietveld refinement indicates that Nb_2_CTe_2_ constitutes 78.0 wt.% of the synthesis products, while Nb_6_C_5_ and NbTe_2_ are present as secondary phases with weight fractions of 15.5 wt.% and 6.5 wt.%, respectively. The reported synthesis conditions were selected as they produced the highest Nb_2_CTe_2_ phase fraction, whereas different temperatures, dwell times, and precursor stoichiometries resulted in reduced phase purity, as summarized in Table 3 and shown in Figure S1. We further note that comparable phase compositions were obtained across repeated syntheses performed under the optimized conditions.

**Table 3:** Phase Compositions of Nb–C–Te Products Obtained under Different Synthesis Temperatures, Dwell Times, and Precursor Stoichiometries^a^

Temperature (°C)	Dwell time (h)	Precursors atomic ratio (Nb:C:Te at.%)	Nb_2_CTe_2_ wt.%	Nb_6_C_5_ wt.%	NbTe_2_ wt.%	Nb_3_Te_4_ wt.%	Nb_2_Te_4_O_13_ wt.%
1100	2	2:1:2	—	6.7	—	93.3	—
1200	2	2:1:2	**78**	15.5	6.5	—	—
1250	2	2:1:2	**35.1**	18.7	46.2	—	—
1200	1	2:1:2	**27.2**	9.9	56.6	—	6.3
1200	3	2:1:2	**47.9**	16.1	36.0	—	—
1200	2	2.2:1:1.8	**42.6**	10.6	8.3	38.5	—
1200	2	1.8:1:2.2	—	16.3	81.2	—	2.5
1200	2	1.8:1:1.8	**68.4**	16.5	15.1	—	—

a The corresponding XRD patterns are shown in Figure S1.

The persistent formation of Nb_6_C_5_ and NbTe_2_ secondary phases under the optimized synthesis conditions reflects the strong thermodynamic stability of both these phases.^27,28^ This interpretation is supported by the synthesis optimization results, which reveal a relatively narrow processing window for Nb_2_CTe_2_ formation. Modest variations in temperature, dwell time, or precursor stoichiometry resulted in substantial reductions in the Nb_2_CTe_2_ phase fraction (Table 3). Notably, extending the dwell time beyond the optimized condition did not improve phase purity, confirming that the residual Nb_6_C_5_ and NbTe_2_ phases are not simply the result of an incomplete reaction. Rather, the results indicate that Nb_2_CTe_2_ formation occurs in competition with energetically favorable carbide and telluride phases. While the conditions investigated in the present study yielded Nb_2_CTe_2_ as the predominant phase (up to 78 wt %), the existence of a narrower synthesis window capable of further suppressing these competing phases cannot be excluded.

We note in passing that Nb_2_CTe_2_ was also synthesized via an alternative two-step route. In this approach, NbTe_2_ is first synthesized by reacting elemental Nb and Te powders in a sealed quartz ampule under vacuum at elevated temperature, yielding NbTe_2_. The resulting NbTe_2_ powder is subsequently used as a less volatile Te source which is then reacted with elemental Nb and C to form Nb_2_CTe_2_. This reaction can be carried out in a conventional tube furnace under flowing Ar, enabling synthesis on a larger batch scale compared to the sealed-ampule route. This alternative approach resulted in Nb_2_CTe_2_ with similar phase purity and morphology to that obtained via the direct elemental synthesis.

SEM micrographs illustrating the morphology of the synthesized Nb_2_CTe_2_ powder are presented in Figure 2. A low-magnification micrograph (Figure 2a) shows the synthesized powder in the form of agglomerated, irregularly shaped particles. Examination at higher magnification (Figure 2b) reveals that the agglomerates consist of fine flake-like particles with well-defined lateral dimensions (up to ∼500 nm). These particles exhibit a lamellar morphology, with individual platelet thicknesses on the order of several tens of nanometers. The observed flake-like morphology is consistent with that reported for TMCC phases^11^ and other layered vdW materials.^29^ A comparative overview of the experimentally reported TMCC phases, including synthesis conditions, structural characteristics, morphology, and exfoliation feasibility, is provided in Table S1.

**Figure 2:**
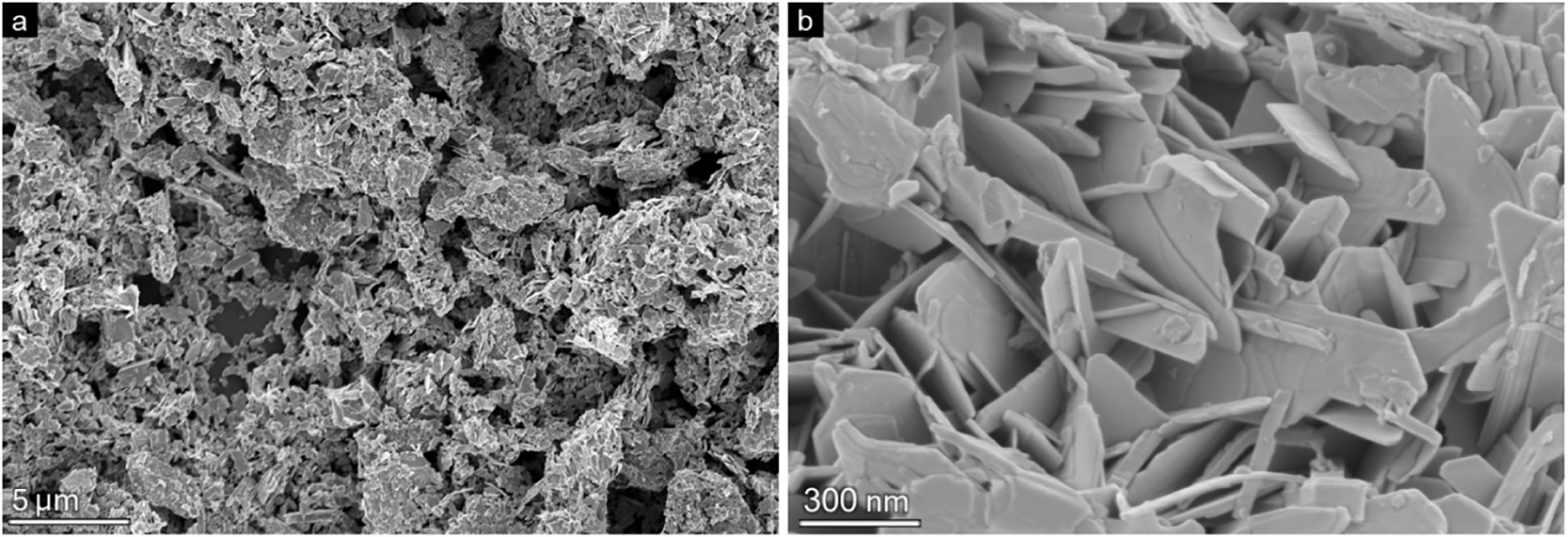
SEM micrographs of the synthesized Nb_2_CTe_2_ powder. (a) Low-magnification micrograph showing agglomerated powder particles. (b) High-magnification micrograph revealing flake-like particles with a lamellar morphology and typical thicknesses on the order of several tens of nanometers.

To further verify the formation of the Nb_2_CTe_2_ TMCC phase, the synthesized material was examined using high-resolution TEM and HAADF-STEM, as shown in Figure 3. A representative flake-like particle identified in the TEM micrograph (Figure 3a) exhibiting the morphology and composition expected for Nb_2_CTe_2_ was subsequently selected for atomic-scale analysis. HAADF-STEM imaging viewed along the [100] zone axis (ZA) reveals a distinct layered atomic arrangement (Figure 3b), which corresponds to the same viewing direction of the crystal structure illustration presented in Figure 1. The corresponding fast Fourier transform (FFT) exhibits well-defined diffraction spots, consistent with the trigonal symmetry determined by XRD. Figure 3b further displays alternating high- and low-intensity atomic planes along the stacking direction. The intensity contrast arises from the atomic number (Z) difference between Te and Nb. This is further supported by the corresponding EDS analysis shown in Figure 3c and the overlaid integrated Nb and Te intensities (Figure 3b), reflecting the periodic stacking of Te and Nb–C layers. Quantitative analysis of the corresponding region in Figure 3c yielded 38.1 at.% Nb,31.1 at.% Te, and 30.8 at.% C (Figure S2 and Table S2). Minor deviation from the ideal stoichiometry is attributed to the relatively thick analyzed lamella region, and contribution from contaminations such as the carbon coating deposited during FIB preparation.

**Figure 3:**
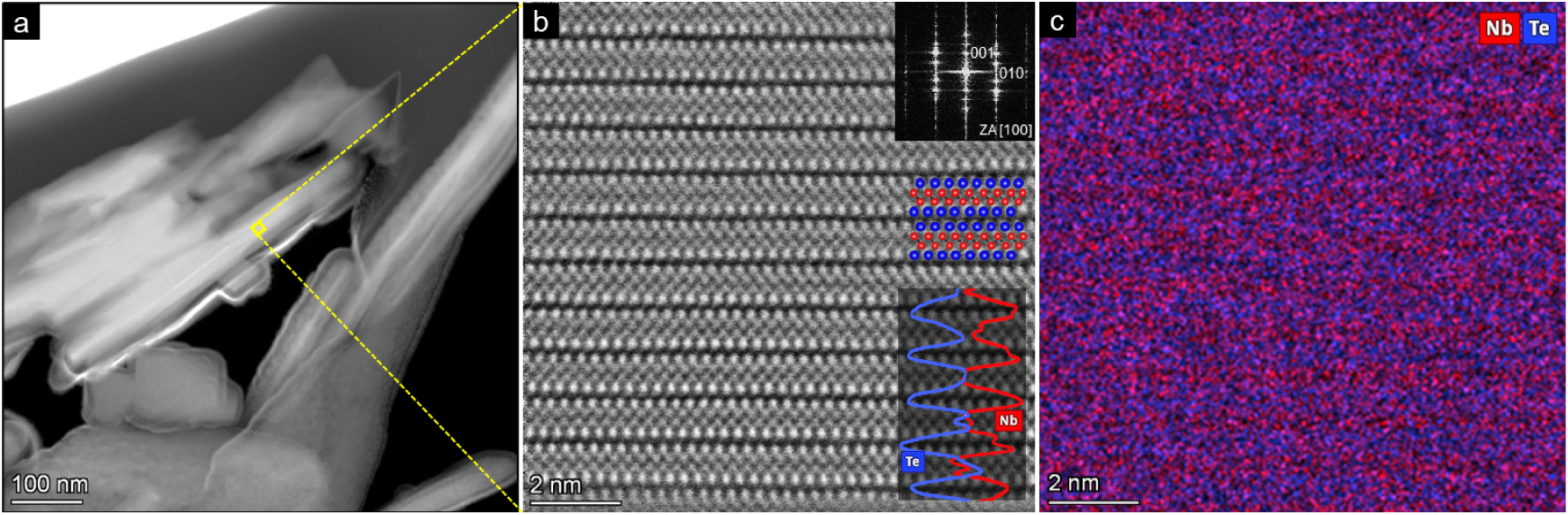
Atomic-scale structural and chemical characterization of the Nb_2_CTe_2_ TMCC phase. (a) TEM micrograph showing a representative flake-like particle selected for atomic-scale analysis. (b) HAADF-STEM micrograph acquired along the [100] ZA, with the corresponding FFT and overlaid with representative atomic schematic and integrated EDS line scan profiles where Nb and Te are represented in red and blue, respectively. (c) The corresponding STEM-EDS elemental analysis of Nb (red) and Te (blue) highlighting the alternating stacking of Nb–C and Te layers.

Further analysis of the HAADF-STEM atomic resolution data was performed to examine the layered structure of Nb_2_CTe_2_. Adjacent Nb_2_CTe_2_ slabs are separated by a low-intensity region. Direct measurements from the HAADF-STEM micrograph (Figure 3b), with the interlayer separation defined as the center-to-center distance between adjacent Te peaks,^30^ indicate an average slab thickness of 0.617 ± 0.054 nm and an interlayer separation of 0.358 ± 0.040 nm, corresponding to approximately 58% of the slab thickness (Figure 4). The presence of a pronounced separation between neighboring slabs, with an interlayer Te–Te distance substantially larger than the expected Te–Te covalent bond length, is consistent with weak interlayer interactions.^31^ Notably, the measured interlayer separation is comparable to Te–Te vdW separations previously reported.^32^ The predicted delamination energy of ∼56 meV/atom for Nb_2_CTe_2_ ^15^ is comparable to the values reported for experimentally exfoliated TMCC phases.^1^ Together with the established correlation between low exfoliation energies and vdW layered structures,^16^ these results further support predominantly weak vdW interlayer coupling.

**Figure 4:**
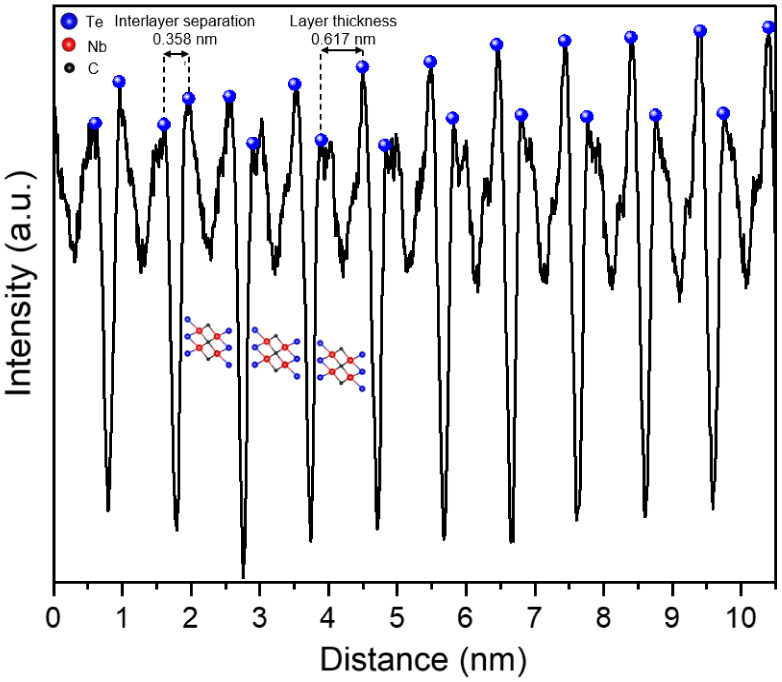
Averaged HAADF intensity profile measured parallel to the Nb_2_CTe_2_ stacking direction, extracted from the micrograph presented in Figure 3b. Blue markers indicate Te atomic-plane intensity maxima used to determine the slab thickness and interlayer separation. The Nb_2_CTe_2_ slab thickness was measured to be 0.617 ± 0.054 nm, while the interlayer separation was 0.358 ± 0.040 nm, corresponding to an interlayer separation of 58% relative to the Nb_2_CTe_2_ slab thickness.

The long-term chemical stability of Nb_2_CTe_2_ was evaluated after prolonged exposure to ambient conditions. Figure 5 shows the diffraction patterns and corresponding Rietveld-refined phase compositions for the as-synthesized material, followed by measurements of the same sample after 10 and 16 months of ambient exposure. As previously discussed, Nb_2_CTe_2_ is the dominant phase in the as-synthesized sample, accounting for 78.0 wt %, together with Nb_6_C_5_ and NbTe_2_ at 15.5 and 6.5 wt %, respectively. After 10 months, the characteristic Nb_2_CTe_2_ peak intensities decrease substantially, and the Nb_2_CTe_2_ phase fraction decreases to 11.4 wt % and further down to 4.3 wt % after 16 months. Correspondingly, the remaining phase fractions become dominated by Nb_6_C_5_ and NbTe_2_, which after 16 months account for 67.1 and 28.6 wt %, respectively.

**Figure 5:**
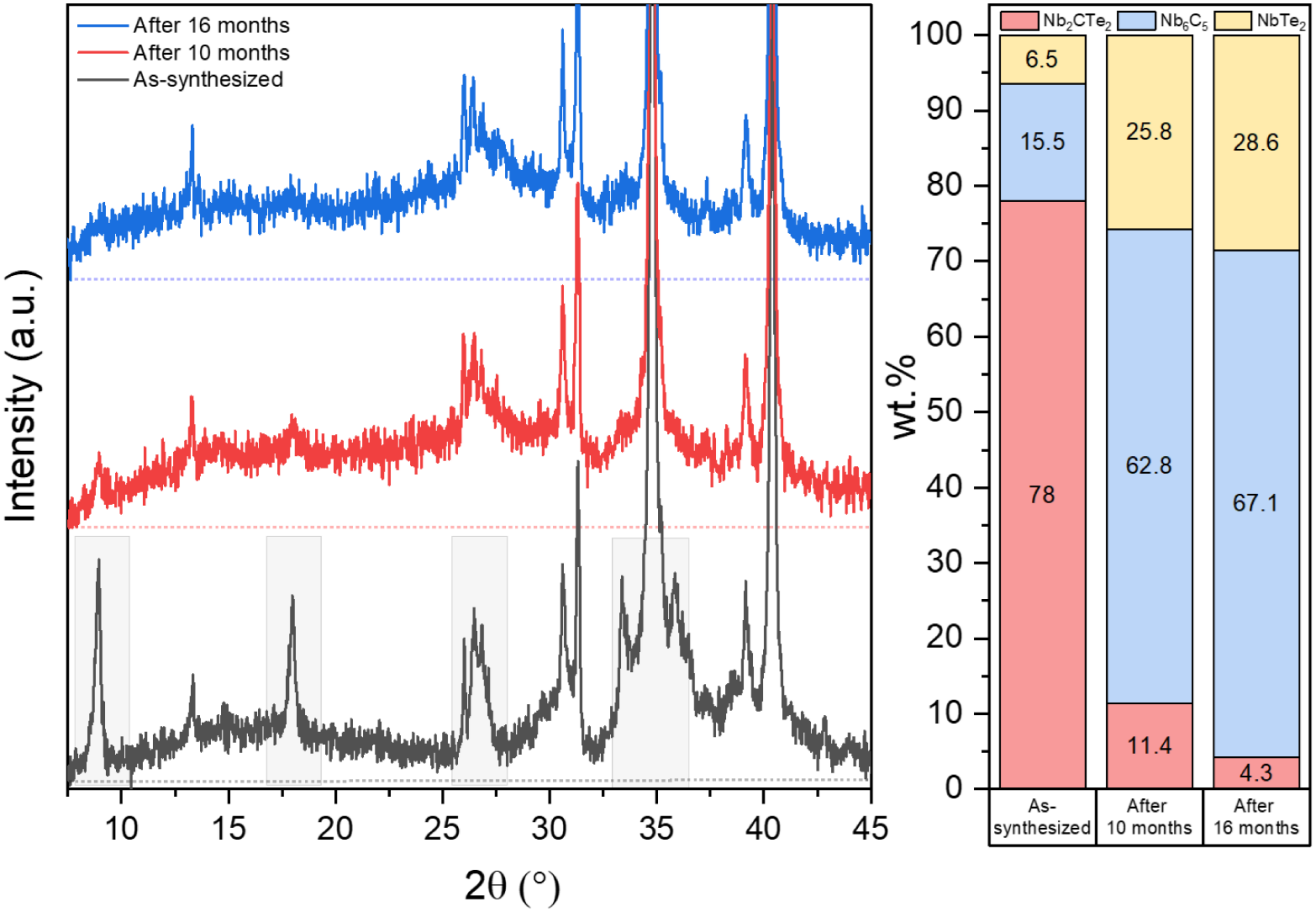
Evolution of Nb_2_CTe_2_ under ambient exposure. XRD patterns collected from the as-synthesized material as well as the same sample after 10 and 16 months are shown together with the corresponding Rietveld-refined crystalline phase compositions. Dashed baselines beneath the diffraction patterns emphasize the increase in the broad diffuse scattering contribution. Gray boxes highlight Nb_2_CTe_2_ reflections, showing their progressive decrease with exposure time.

In addition to the changes in the crystalline phase composition, the diffraction patterns of the aged samples exhibit broad diffuse scattering (emphasized by the colored dashed baselines beneath the diffraction patterns in Figure 5). This suggests that the degradation/oxidation process involves formation of amorphous phases. A quantitative estimate of the amorphous contribution was therefore obtained using AMORPH^25^ analysis over the 20–50° region. The fitted relative amorphous contribution shows a clear trend, increasing from <1 wt % in the as-synthesized sample to 26.7 wt % after 10 months and 35.8 wt % after 16 months. This is further supported by high-resolution SEM-EDS analysis of the aged material (Figures S3–S4 and Table S3), which shows oxygen distribution over the particles. The observed susceptibility to ambient degradation is consistent with the thin flake-like morphology (Figure 2b), where particle thicknesses on the order of tens of nanometers provide a large, exposed surface area. Furthermore, the aged patterns contain some unresolved features that are not represented as distinct crystalline phases in the Rietveld refinement and may be associated with degradation/oxidation products (see Figure S5). These results support progressive, oxidation-associated degradation in Nb_2_CTe_2_ under ambient exposure, consistent with prior reports of ambient oxidation and long-term degradation in other Te-based compounds^33–35^ and TMDs.^36^

## Conclusions

This study reports the experimental realization of Nb_2_CTe_2_, representing the first tellurium-based TMCC phase. Combined XRD, high-resolution SEM, and atomic-resolution STEM-EDS analyses confirm that Nb_2_CTe_2_ was predominantly obtained via reactive synthesis under optimized conditions of 1200 °C for 2 h. Nb_2_CTe_2_ crystallizes in a trigonal structure with *P*3̅*m*1 symmetry and forms flake-like particles with thicknesses on the order of several tens of nanometers. Structurally, Nb_2_CTe_2_ consists of Nb–C carbide slabs capped by Te layers, forming a layered structure in which adjacent slabs are separated by an interlayer gap of ∼0.358 nm. This pronounced spacing, together with the previously predicted low delamination energy of Nb_2_CTe_2_, supports weak out-of-plane vdW coupling between adjacent slabs. Nb_2_CTe_2_ exhibited limited long-term chemical stability under ambient conditions, undergoing oxidative degradation over time with near-complete loss after 16 months in air. The successful synthesis of Nb_2_CTe_2_ expands the experimentally realized TMCC family and establishes a foundation for the development of two-dimensional Nb_2_CTe_2_ derivatives.

## Supplementary Material





## Notes

Crystallographic information file for Nb_2_CTe_2_ (2521070) (CIF)
XRD analysis of synthesis optimization; comparison of experimentally reported TMCC phases; supplementary STEM-EDS characterization; SEM–EDS and Rietveld analyses after ambient exposure (PDF)
