# Supplementary material for: Extending Transition-Metal Carbo-Chalcogenides to Tellurium with Nb2CTe2
Source: ACS Omega. 2026 Jul 29;11(31):46136–42. doi: 10.1021/acsomega.6c02686 (PMC13470694; doi:10.1021/acsomega.6c02686)
Supplement: Supplementary file 1 [file ao6c02686_si_002.pdf]

Supplementary information

Extending Transition-Metal Carbo-Chalcogenides to Tellurium with Nb_2CTe_2

Yiftach Kushnir[a], Mark Baranov[b], Bar Favelukis[a], Asaf Nitsan[a], Or Messer[a], Alexander Upcher[b], Vladimir Ezersky[b], Nitzan Maman[b], Barak Ratzker[a,c], Maxim Sokol[a,*]

[a] *Department of Materials Science and Engineering, Tel Aviv University, P.O.B 39040, Ramat Aviv 6997801, Israel*

[b] *Ilse Katz Institute for Nanoscale Science and Technology, Ben-Gurion University of the Negev, P.O.B 653, Beer-Sheva 8410501, Israel*

[c] *Max Planck Institute for Sustainable Materials, Max-Planck-Str. 1, 40237 Düsseldorf, Germany*

[*] Corresponding author: sokolmax@tauex.tau.ac.il

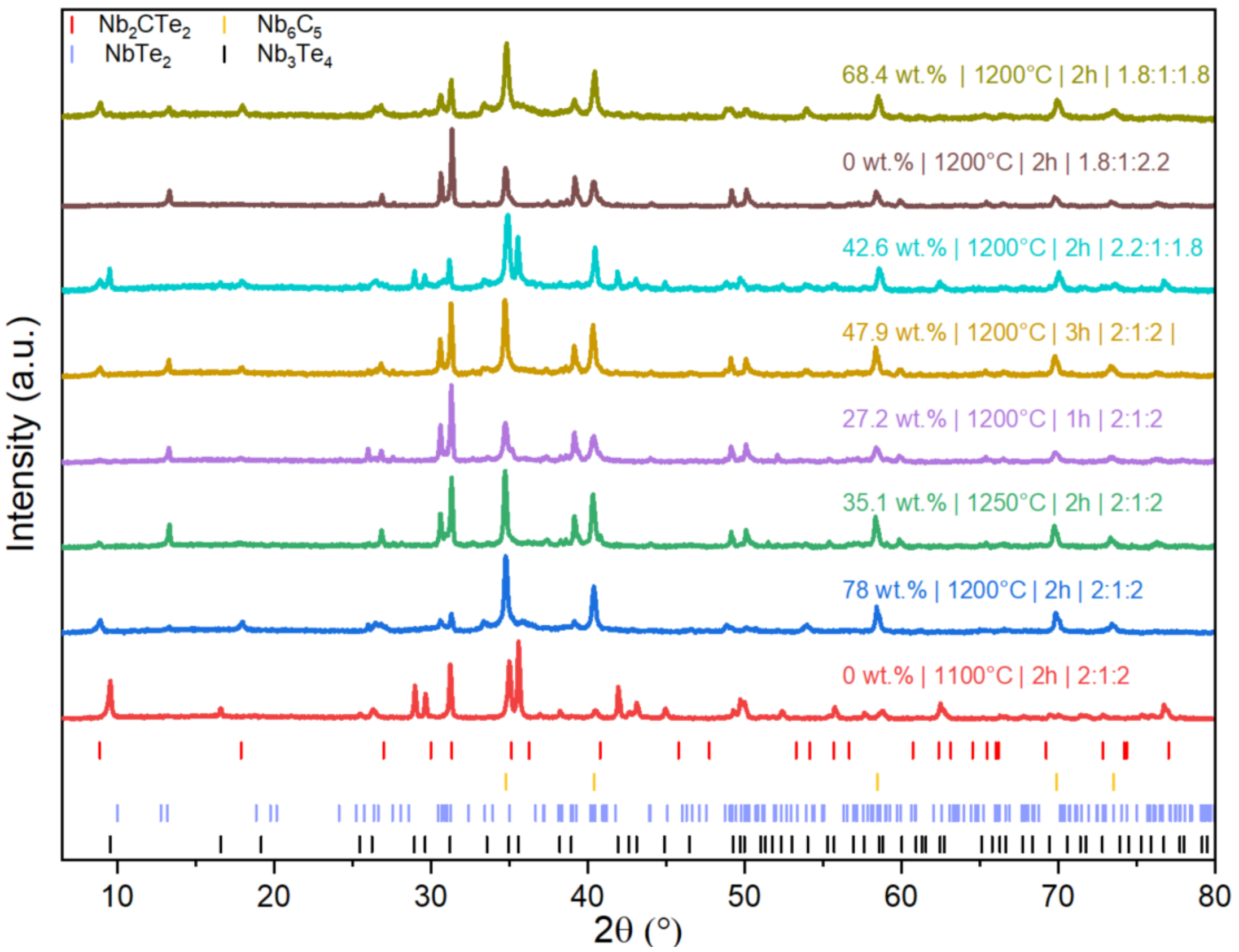

Figure S1. XRD patterns of Nb_2CTe_2 synthesis products obtained under different precursor atomic ratios and thermal treatment conditions, corresponding to the results summarized in Table 3. The legend associated with each pattern indicates the refined Nb_2CTe_2 phase fraction (wt.%), synthesis temperature, dwell time and starting Nb:C:Te atomic ratio. The highest Nb_2CTe_2 phase fraction was obtained for the 2:1:2 1200 °C 2 h synthesis; peak identification for this synthesis is presented in Figure 1.

Table S1. Comparison of the structural parameters, synthesis conditions, and exfoliation behavior of experimentally reported TMCC phases.

TMCC phase	Space group symmetry	*a* (Å)	*c* (Å)	Synthesis Method	Synthesis temperature (°C)	Quantitative phase analysis (wt.%)	Morphology	Exfoliation feasibility	Calculated Delamination Energy (meV/atom)	Reference
Ta_2CS_2	$P\bar{3}m1$ [(a)]	3.265	8.537	Elemental solid-state synthesis, Topochemical synthesis (deintercalation of Fe)	1200-1550	Not reported	Layered/ flake-like	Experimentally demonstrated	~46.61	[1–3]
Nb_2CS_2	$P\bar{3}m1$ [(a)]	3.270	8.560	Topochemical synthesis (deintercalation of Cu)	1000-1200	Not reported	Layered/ flake-like	Experimentally demonstrated	~51.34	[1,3,4]
Nb_2CSe_2	$P\bar{3}m1$	3.315	8.997	solid-state vapor assisted synthesis	1200	99	Layered/ flake-like	Predicted	~51.37	[1,5,6]
Ta_2CSe_2	$P\bar{3}m1$	3.290	8.930	Topochemical synthesis (deintercalation of Fe)	1200	93	Layered/ flake-like	Experimentally demonstrated	~50.16	[1,7]
Nb_2CTe_2	$P\bar{3}m1$	3.483	9.884	Elemental solid-state synthesis	1200	78	Layered/ flake-like	Predicted	~55.74	[1], this work

(a) Ta_2CS_2 and Nb_2CS_2 have also been reported in a rhombohedral 3R polymorph (space group *R*3*m*, #160**)** [2,3]**.**

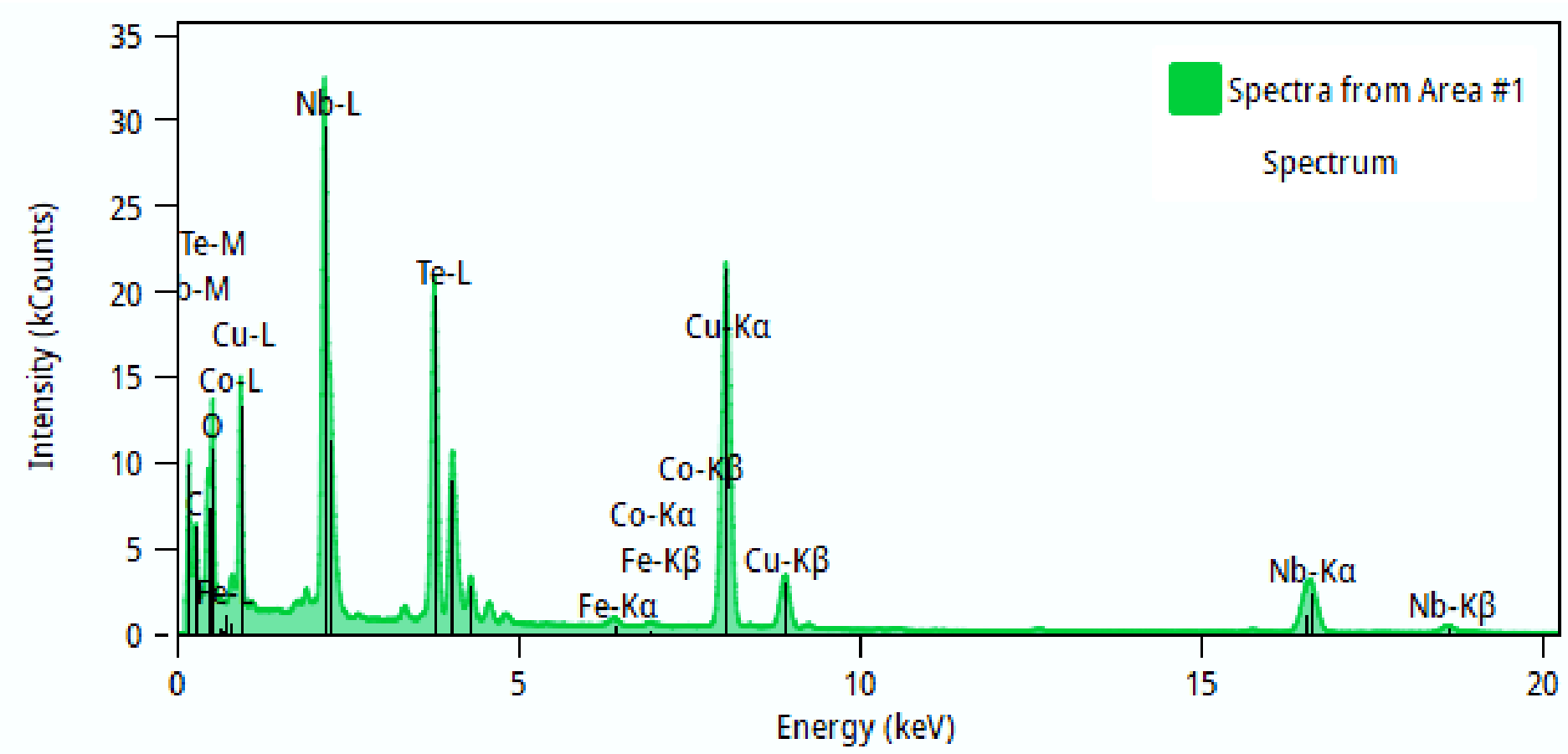

Figure S2. EDS spectrum obtained for the region shown in Figure 3c; the corresponding at.% values are given in Table S2.

Table S2. EDS atomic fraction quantification of C, Nb, and Te corresponding to the region measured in Figure 3c, showing the ratio of Nb:Te being roughly 1.23:1.

Element	Z	Family	Atomic fraction (%)
C	6	K	30.79 ± 1.85
Nb	41	K	38.11 ± 3.30
Te	52	L	31.09 ± 2.82

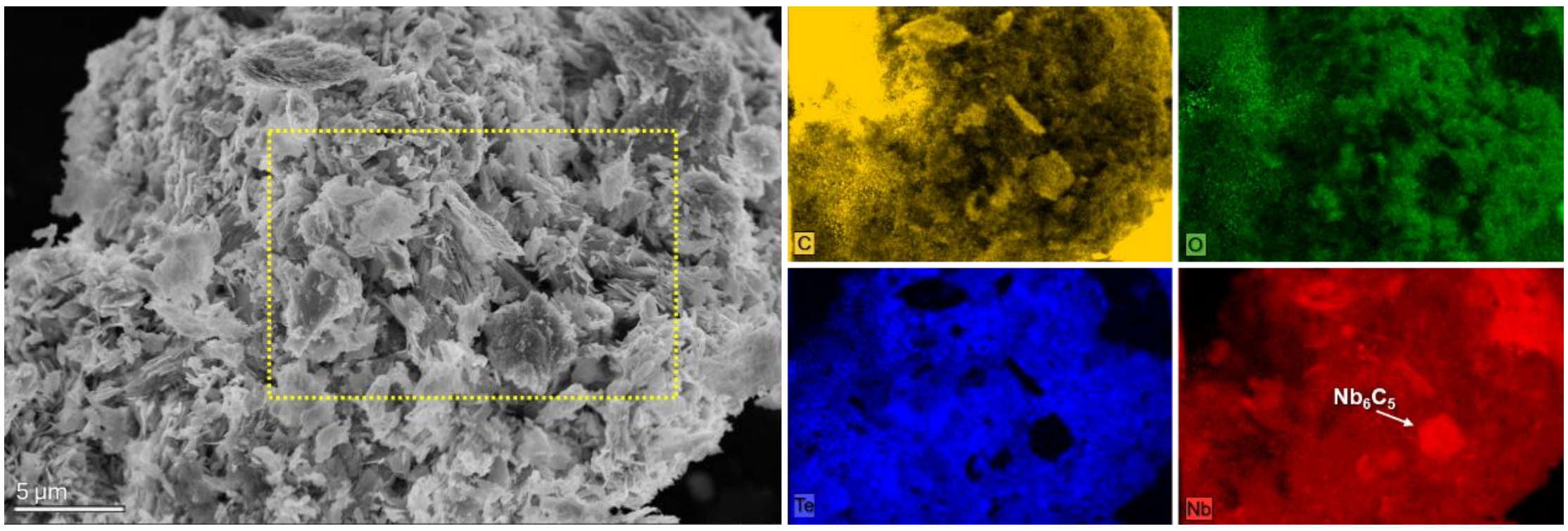

Figure S3. SEM-EDS analysis of the Nb_2CTe_2 powder after 16 months of ambient exposure. SEM micrograph showing the agglomerated particle morphology, with the yellow dashed rectangle marking the region used for quantitative EDS analysis in Figure S4. Corresponding elemental maps show the spatial distribution of C (yellow), O (green), Te (blue), and Nb (red). The Nb_6C_5 region is indicated in the Nb map; $NbTe_2$ could not be spatially resolved due to the limited C-map resolution.

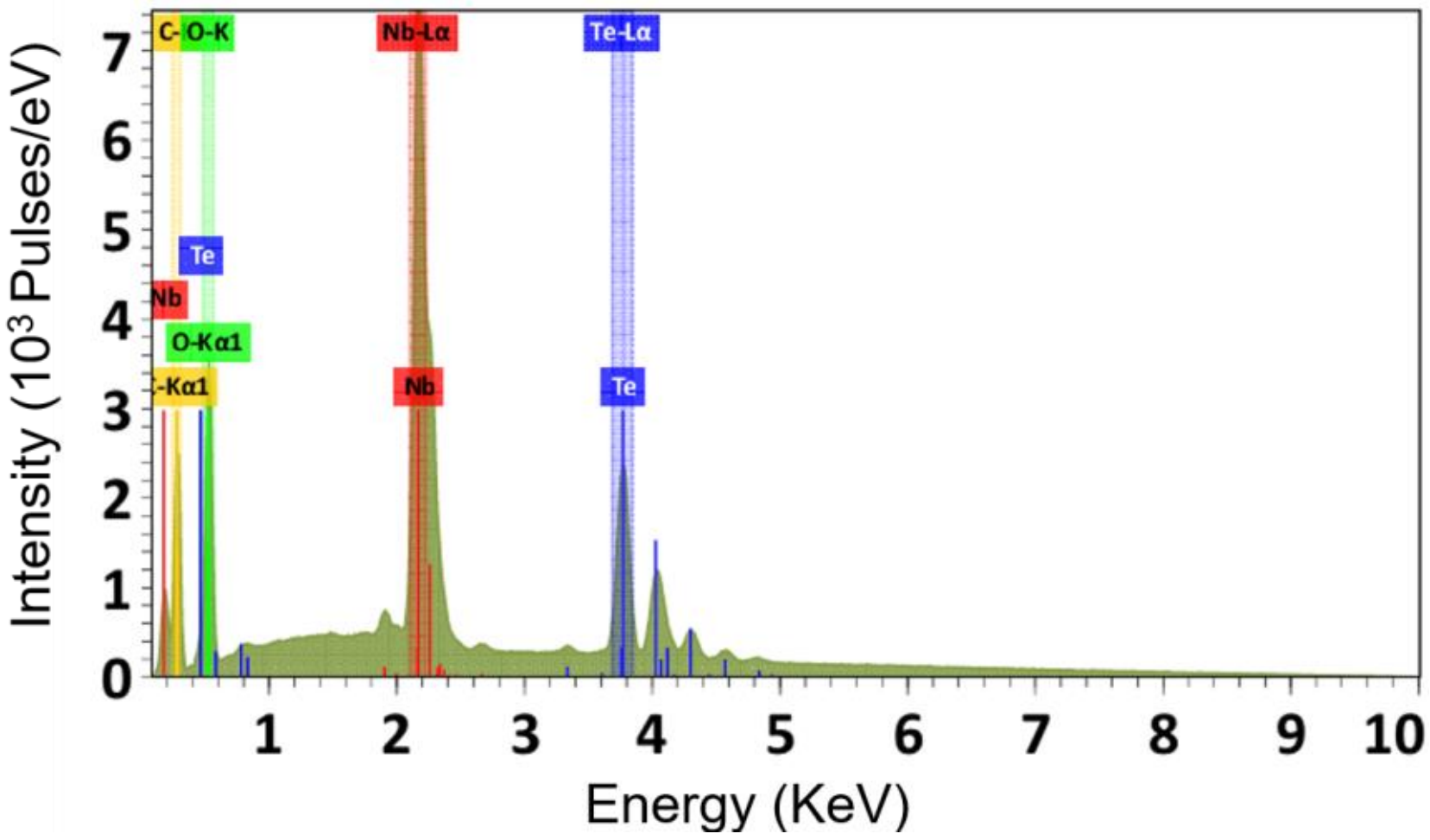

Figure S4. EDS spectrum obtained for the region shown in Figure S3; the corresponding at.% values are given in Table S3.

Table S3. EDS atomic fraction quantification of C, O, Nb and Te corresponding to the region measured in Figure S3 and displayed in Figure S4.

Element	Z	Family	Atomic fraction (%)
C	6	K	42.56 ± 4.60
O	8	K	22.33 ± 2.44
Nb	41	K	21.71 ± 0.80
Te	52	L	13.40 ± 0.45

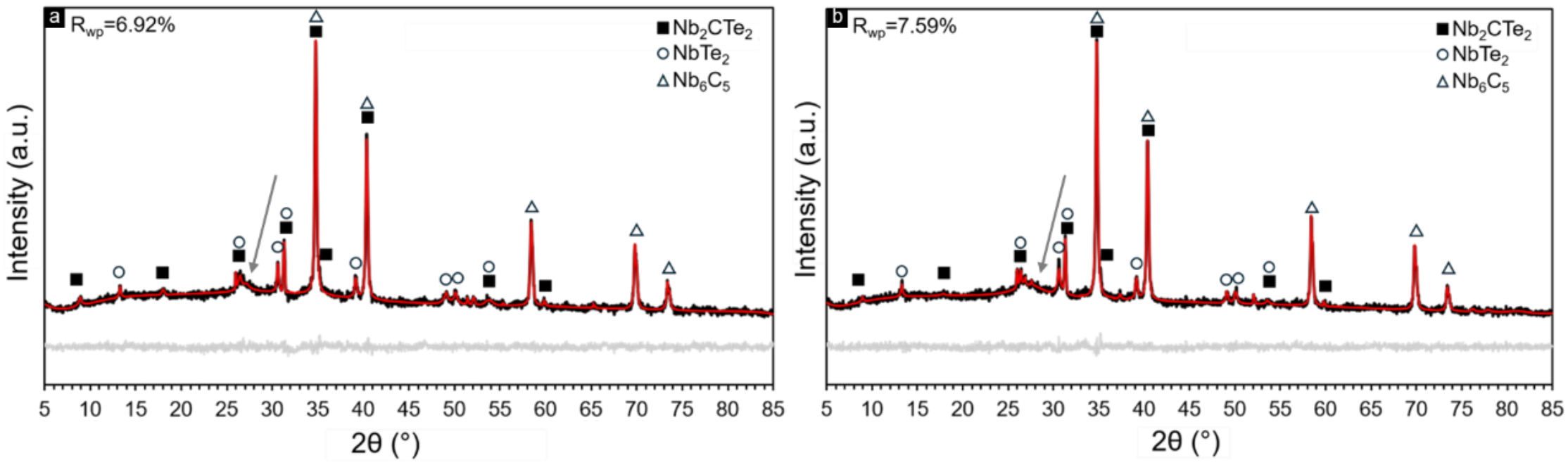

Figure S5. Rietveld refinements of the Nb_2CTe_2 powder after ambient exposure for (a) 10 months and (b) 16 months. The refined crystalline phase composition is 11.4 wt.% Nb_2CTe_2, 25.8 wt.% $NbTe_2$, and 62.8 wt.% Nb_6C_5 after 10 months, and 4.3 wt.% Nb_2CTe_2, 28.6 wt.% $NbTe_2$, and 67.1 wt.% Nb_6C_5 after 16 months. The refinements converged to R_{wp} = 6.92% and 7.59%, respectively. Gray arrows highlight weak, unresolved diffraction features that are not represented as distinct crystalline phases in the refinement.

References

[1] Helmer P, Björk J, Rosen J. Computational Screening of Chalcogen-Terminated Inherent Multilayer MXenes and M_2AX Precursors. *Inorg Chem.* 2024. DOI: 10.1021/acs.inorgchem.4c01690

[2] Beckmann O, Boller H, Nowotny H. The Crystal Structures of Ta_2S_2C and Ti_4S_5 ($Ti_{0,81}S$). Monatshefte Für Chem. 1970;101(4):945–955. DOI: 10.1007/BF00908535

[3] Majed A, Kothakonda M, Wang F, et al. Transition Metal Carbo-Chalcogenide "TMCC:" A New Family of 2D Materials. *Adv Mater*. 2022;34(26):2200574. DOI: 10.1002/adma.202200574

[4] Boller H, Hiebl K. Quaternary pseudo-intercalation phases $T_x[Nb_2S_2C]$ (T=V, Cr, Mn, Fe, Co, Ni, Cu) and metastable Nb_2S_2C formed by topochemical synthesis. *J. Alloys Compd.* 1992;183:438–443. DOI: 10.1016/0925-8388(92)90765-2

[5] Pang X, Wu T, Gu Y, et al. Nb_2Se_2C: a new compound as a combination of transition metal dichalcogenide and MXene for oxygen evolution reaction. *Chem. Commun.* 2020;56(63):9036–9039. DOI: 10.1039/D0CC03282D

[6] Pang X, Lv Z, Xu S, et al. Ultra-conductive Se-terminated MXene Nb_2CSe_2 via one-step synthesis for flexible fast-charging batteries. *Energy Storage Mater.* 2023;61:102860. DOI: 10.1016/j.ensm.2023.102860

[7] Loni E, Majed A, Zhang S, et al. Two-Dimensional Tantalum Carbo-Selenide for Hydrogen Evolution. *ACS Nano*. 2025 DOI: 10.1021/acsnano.4c09903.
